# Characterizing phonemic fluency by transfer learning with deep language models

**DOI:** 10.1093/braincomms/fcad318

**Published:** 2023-11-28

**Authors:** Joe Mole, Amy Nelson, Edgar Chan, Lisa Cipolotti, Parashkev Nachev

**Affiliations:** Department of Neuropsychology, National Hospital for Neurology and Neurosurgery, London WC1N 3BG, UK; Institute of Neurology, University College London, London WC1N 3BG, UK; Institute of Neurology, University College London, London WC1N 3BG, UK; Department of Neuropsychology, National Hospital for Neurology and Neurosurgery, London WC1N 3BG, UK; Institute of Neurology, University College London, London WC1N 3BG, UK; Department of Neuropsychology, National Hospital for Neurology and Neurosurgery, London WC1N 3BG, UK; Institute of Neurology, University College London, London WC1N 3BG, UK; Institute of Neurology, University College London, London WC1N 3BG, UK

**Keywords:** fluency, frontal lobes, machine learning, language modelling, executive functions

## Abstract

Though phonemic fluency tasks are traditionally indexed by the number of correct responses, the underlying disorder may shape the specific choice of words—both correct and erroneous. We report the first comprehensive qualitative analysis of incorrect and correct words generated on the phonemic (‘S’) fluency test, in a large sample of patients (*n* = 239) with focal, unilateral frontal or posterior lesions and healthy controls (*n* = 136). We conducted detailed qualitative analyses of the single words generated in the phonemic fluency task using categorical descriptions for different types of errors, low-frequency words and clustering/switching. We further analysed patients’ and healthy controls’ entire sequences of words by employing stochastic block modelling of Generative Pretrained Transformer 3–based deep language representations. We conducted predictive modelling to investigate whether deep language representations of word sequences improved the accuracy of detecting the presence of frontal lesions using the phonemic fluency test. Our qualitative analyses of the single words generated revealed several novel findings. For the different types of errors analysed, we found a non-lateralized frontal effect for profanities, left frontal effects for proper nouns and permutations and a left posterior effect for perseverations. For correct words, we found a left frontal effect for low-frequency words. Our novel large language model–based approach found five distinct communities whose varied word selection patterns reflected characteristic demographic and clinical features. Predictive modelling showed that a model based on Generative Pretrained Transformer 3–derived word sequence representations predicted the presence of frontal lesions with greater fidelity than models of native features. Our study reveals a characteristic pattern of phonemic fluency responses produced by patients with frontal lesions. These findings demonstrate the significant inferential and diagnostic value of characterizing qualitative features of phonemic fluency performance with large language models and stochastic block modelling.

## Teaser

Mole, Nelson and colleagues report on the largest analysis of the quality of phonemic fluency responses in focally lesioned patients. A novel transfer learning approach, capable of identifying left frontal patients based on quality of responses, is discussed. This approach predicted frontal dysfunction with far greater accuracy than other features.

## Graphical Abstract

**Graphical Abstract:**
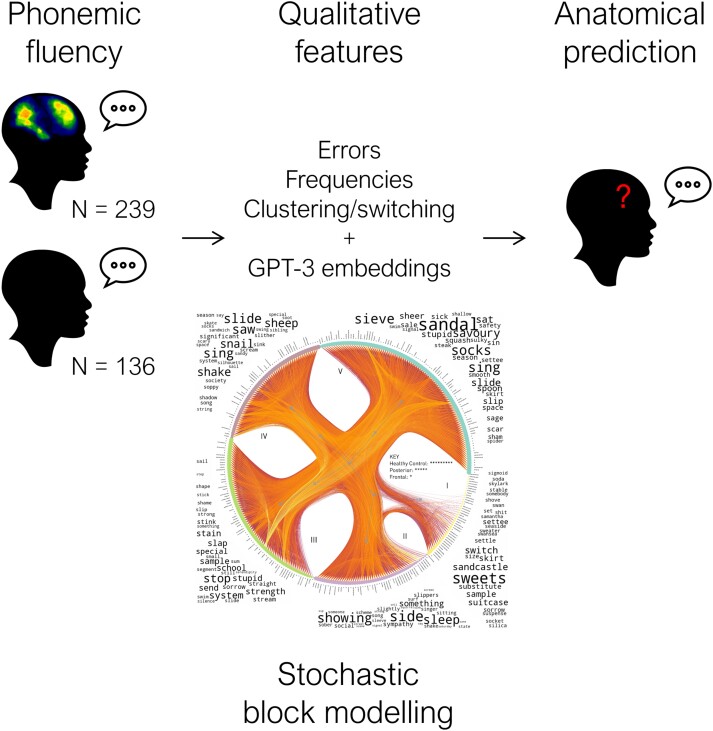

## Introduction

Our understanding of the human brain is constrained by the need to reduce complex behaviour to that which is measurable. One approach to achieving this is to measure cognitive abilities with a single metric, such as the score on a cognitive test. While this approach has obvious advantages, not least simplicity, it leaves a wealth of potentially informative aspects of performance unexploited.

This issue is of central importance to studies of the effects of focal brain damage on cognitive performance, where the analysis of the ‘quality’, not just ‘quantity’, of responses provides a unique form of evidence not available to other cognitive neuroscience methods.^1,2^ This methodology is held to be of critical theoretical value, providing a unique means of investigating how cognitive functions are organized. It is also thought to be clinically useful, as a diagnostic marker of specific neurocognitive dysfunction. Yet, relatively few focal lesion group studies have conducted a systematic analysis of the ‘quality’ of patients’ responses on some of the most widely used cognitive tests, such as the phonemic fluency test.

The phonemic fluency test is among one of the most well-established measures of frontal lobe function.^3^ On these tests, patients are required to generate a series of words beginning with the same phoneme within 60 s, without producing proper nouns, permutations of previous words or repetitions. Like most frontal tasks, these tests are thought to draw upon different complex executive processes whose nature is still incompletely understood. Several studies analysing overall S performance (total number of correct words generated) have shown that phonemic fluency is a task that shows specificity to frontal lesions, with frontal patients more impaired than posterior patients, and it is lateralized, with left frontal patients significantly more impaired than right frontal patients (e.g.^4-6^).

However, very few studies (<10) have conducted, preliminary, investigations of the incorrect and correct words generated in phonemic fluency tasks by patients and healthy controls (see Cipolotti *et al*.^5^ for a review). Usually, the overall number of errors has been analysed^7^ and conflicting results are reported. For example, some studies reported no difference between frontal patients and healthy controls,^7^ whilst others found significant differences.^4,8^ However, as qualitatively different types of error may result from disruption to different executive processes, summing across error types may overlook potentially informative effects. Two recent studies have investigated two specific types of error: rule break, where the known task rules are violated, and perseveration, where the same words are given more than once. Both studies reported that rule break errors were associated with left frontal lesions and no specific brain localization was found for perseverations.^9,10^ However, rule breaks encompass several different types of errors, such as inappropriate words, proper nouns, permutations of previous words and words beginning with the wrong letter. We recently investigated only one type of rule break error, namely, the generation of inappropriate words, and found that they were associated with frontal, rather than non-frontal, lesions.^5^

Troyer and colleagues^11^ analysed both errors and correct words and reported reduced switching between ‘clusters’ of phonemically similar words in patients with left dorsolateral or superior medial frontal lesions relative to healthy controls. However, other studies that have analysed clustering and switching scores have reported somewhat mixed results. For example, Stuss and colleagues^8^ reported no significant differences between patients with focal left, right or bilateral frontal lesions, left or right non-frontal lesions and healthy controls in terms of mean ‘cluster size’ (two or more words beginning with at least the same first two letters e.g. ‘snore, snail, show’ cluster size =1) or the percentage of clustered words relative to the total number of words produced. In contrast, Babulal and colleagues^12^ reported that, in comparison with healthy controls, both frontal and posterior patients produced significantly smaller clusters of words and switched between clusters significantly less frequently. Davidson and colleagues reported that a small sample of right frontal patients (*n* = 20) switched significantly less frequently than healthy controls but the groups did not significantly differ in terms of average cluster size.^13^ Thus, it remains unclear whether clustering and switching are markers of frontal lobe dysfunction.

A further potentially rich, yet relatively unexplored, source of information comes from analysis of the correct words generated. Preliminary findings suggest that extremely infrequent/unknown words (e.g. ‘salacious’) are more commonly generated by frontal patients.^5^ This is potentially of significant theoretical interest, as it may provide important insights into the frontal mechanisms involved in language generation.

Hence, despite the critical theoretical importance of detailed qualitative analysis of words generated on phonemic fluency tasks, there is a surprising paucity of studies on errors and correct words. As far as we are aware, no focal lesion study has yet conducted a comprehensive analysis of the different types of rule break error and correct words generated.

Notably, these types of qualitative analyses mostly investigate the single words generated in the phonemic fluency task using categorical descriptions for different types of errors and/or correct words. However, this fails to consider another important qualitative aspect, namely that in phonemic fluency tasks the participants generate sequences of words. These are likely to be influenced by several factors other than those subject to categorical description and are difficult to capture with the conventional qualitative methods described above. Word sequences are extraordinary rich, varying across several dimensions, including lexical, phonemic and semantic variables. Moreover, they are likely to be influenced by high-level ‘active thinking’ processes, such as strategy formation, inhibition and selection of responses.^2^ To analyse these complex aspects requires models of flexibility only plausibly achievable with machine learning. This approach uses algorithms to find patterns in complex data sets, such as those relating to language, that are not intuitively discernible and not adequately captured by qualitative analysis focusing on different categorical descriptions of single words generated in the phonemic fluency. This presents an opportunity to model phonemic fluency production at a remarkable level of complexity and more comprehensively than has previously been possible.

However, the application of machine learning to phonemic fluency production is fraught with danger. Increasing model flexibility inevitably raises the minimum scale of data needed to assure a generalizable fit. Applying machine learning within the small-scale data regimes typical of neuropsychological investigations risks overfitting to the training data. This concern has limited recent attempts, focused on patients with non-focal neurological damage, such as dementia,^14-18^ to comparatively simple, often manually derived characterizations of individual words taken in isolation. To the best of our knowledge, entire word sequences have so far been left unmodelled in patients with focal lesions.

The aim of this paper is to produce the first comprehensive qualitative analysis in the largest data set of words generated by patients with focal, unilateral, frontal (*n* = 143) or posterior lesions (*n* = 96) and healthy controls to date (*n* = 136). We conduct qualitative analyses investigating single words generated using categorical descriptions for different types of rule break error, perseverations, word frequency of the correct words generated and clustering/switching. In addition, we introduce a novel machine learning approach, relying on rich yet compact representations of language—including ‘sequences’ of words, not just words in isolation. Our approach exploits highly expressive, large language models (LLMs) trained on large-scale natural language corpora ‘outside’ our specific phonemic fluency task. Here, we use Generative Pretrained Transformer 3 (GPT-3), at the time of the study the most powerful LLM, with a capacity to embed not just words but long sequences of words and sensitivity to long-range interactions between words. Such ‘transfer learning’ allows us to model a much richer set of language characteristics than the limited scale of available test data ordinarily permits. The unit of analysis here becomes not the individual word but the entirety of the generated sequence. We can thus combine the model flexibility large-scale data permits with the data economy the neuropsychological domain demands, yielding models that can both capture the richness of language and remain tractable in the setting of small-scale data.

We compare our qualitative analyses of single words based on categorical descriptions of errors, word frequency and clustering/switching to our novel transfer learning approach. Such comparison aims to predict the presence or absence of frontal lesions from generated words, quantifying the additional information contained in language representations over fluency scores, and to illuminate further the mechanisms underlying phonemic fluency performance.

## Materials and methods

### Participants

Patients with focal, unilateral frontal or posterior brain lesions, who attended the Neuropsychology Department of the National Hospital for Neurology and Neurosurgery (Queen Square, London, UK), were retrospectively evaluated for eligibility. Inclusion criteria were (i) presence of a stroke or brain tumour; (ii) ≥70% of the total lesion in the frontal or posterior areas (see Neuroimaging section); (iii) age between 18 and 80 years; (iv) no gross language impairments, i.e. no dysphasic patients were included in this study [no agrammatism, >5th %ile on the Graded Difficulty Naming Test (GNT);^19^ for the few patients where GNT data were not available, performance on the Oldfield Naming test, a score of >24/30 was considered intact],^20,21^ nor perceptual impairments (>5th cut-off on the Incomplete Letters test);^22^ (v) absence of psychiatric disorders, history of alcohol or substance abuse or previous neurological disorders; (vi) native English speaking; and (vii) availability of data on individual words generated during S fluency.

A total of 239 patients with unilateral, focal lesions met the inclusion criteria for the study. This included patients with unilateral frontal lesions (left, *n* = 63; right, *n* = 80), of which 116 have been previously reported,^5,9^ and posterior lesions (left, *n* = 33; right, *n* = 63; see Table 1). The grouping together of focal patients with different aetiologies for the purposes of examining cognitive variables is a common approach (see Cipolotti *et al*.^9^ for further discussion) and, importantly, is one that we have previously shown is methodologically justifiable.^23,24^

**Table 1:** Demographics and overall S fluency performance

	Frontal			Posterior			HC
	Total	Left	Right	Total	Left	Right	*n* = 136
	*n* = 143	*n* = 63	*n* = 80	*n* = 96	*n* = 33	*n* = 63	
Age (years) (SD)	46.77 (15.17)	44.38 (15.26)	48.65 (14.93)	51.04 (13.47)	50.21 (14.72)	51.48 (12.87)	47.93 (15.38)
Gender (male/female)	77/66	**39/24** b**	40/40	56/40	**23/10** b**	37/26	60/76
Ethnicity (White British/Black African or Caribbean/Asian Indian/other)	114/2/4/17	49/1/2/6	65/1/2/11	74/2/4/6	27/1/0/2	47/1/4/4	-
Aetiology (stroke/tumour/abscess)	**35/106/2** a*	11/51/1	24/55/1	35/61/0	**13/20/0** c*	22/41/0	
Education (years) (SD)	13.88 (3.34)	13.77 (3.41)	13.95 (3.31)	13.51 (3.14)	13.97 (3.17)	13.25 (3.13)	13.77 (2.60)
Lesion volume (mm^3^) (SD)	44.33 (46.64)	48.45 (48.13)	41.15 (45.63)	49.69 (69.00)	**28.60** f* (27.**29)**	60.23 (80.52)	
Premorbid NART IQ (SD)	107.32 (11.38)	105.88 (12.62)	108.42 (10.28)	108.51 (12.26)	109.45 (14.31)	107.96 (11.00)	108.09 (9.80)
GNT (correct/30) (SD)	20.97 (3.78)	20.00 (3.64)	21.43 (3.63)	21.45 (4.17)	21.27 (4.76)	21.54 (3.89)	22.29 (3.57)
Overall S performance (SD)	**12.80** a*** b*** (5.**78)**	**11.40** b*** d** e*** (5.**75)**	**13.90** b*** (5.**59)**	15.42 (4.67)	15.27 (4.54)	15.49 (4.77)	16.67 (4.05)

Scores with significant *P* values are in bold. Ethnicity was unavailable for 32 patients and all healthy controls.

HC, healthy control; *n*, number; SD, standard deviation; NART, National Adult Reading Test; GNT, Graded Difficulty Naming Test.

^a^Significant difference from posteriors.

^b^Significant difference from healthy controls.

^c^Significant difference from left frontal.

^d^Significant difference from right frontal.

^e^Significant difference from left posterior.

^f^Significant difference from right posterior.

*P < 0.05; **P < 0.01; ***P < 0.001.

We also recruited a group of 136 healthy controls, with no neurological or psychiatric history, for comparison with frontal and posterior groups on cognitive variables. The healthy control group was closely matched to the patient sample for age, gender, years of education and estimated premorbid level of function based on the National Adult Reading Test (NART).^25^ Unfortunately, ethnicity was not recorded for participants in the healthy control group but, given that the sample primarily comprised of the friends and family of the patient sample, it is reasonable to expect that they were relatively well matched. The study was approved by the National Hospital for Neurology and Neurosurgery and Institute of Neurology Joint Research Ethics Committee and conducted in accordance with the Declaration of Helsinki.

### Neuroimaging

**Figure 1:**
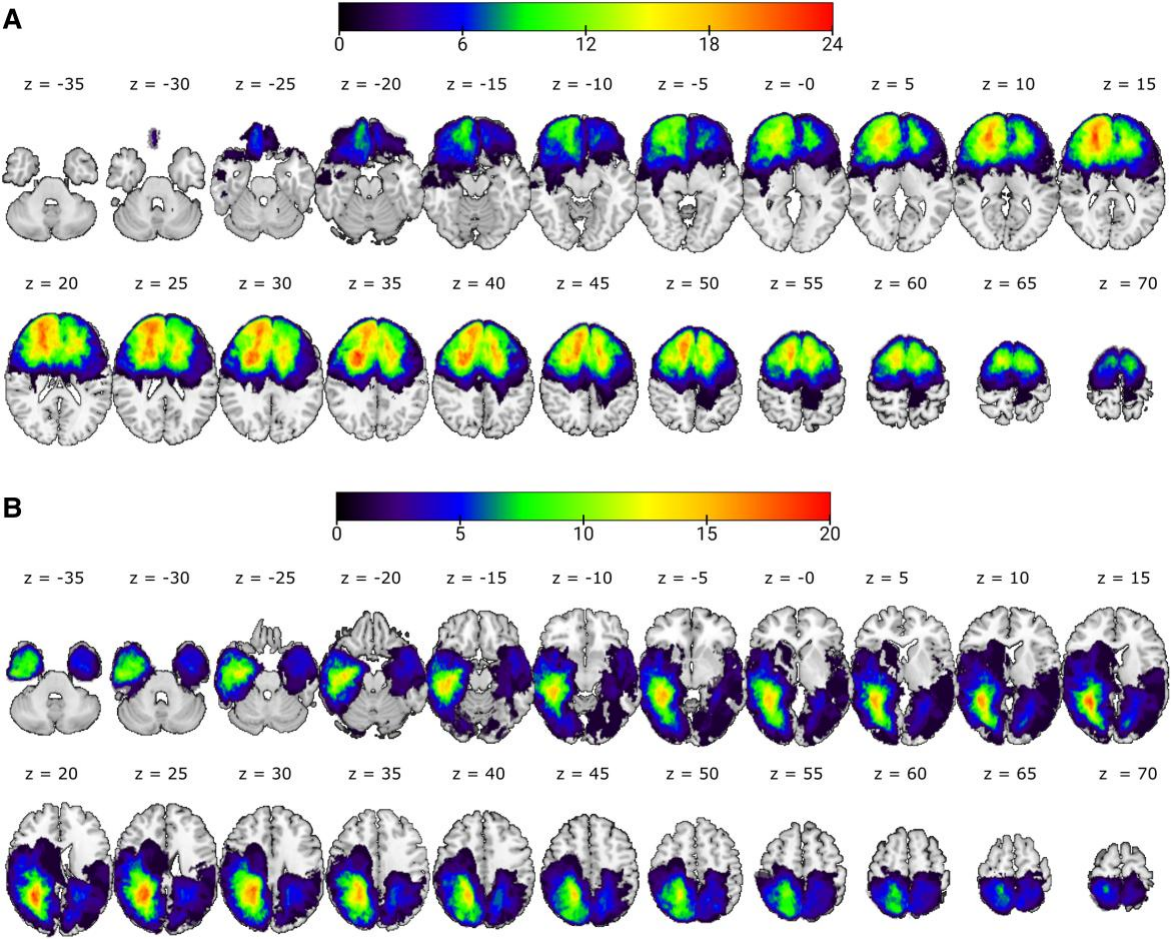
**Lesion distribution volume map for the (A) frontal group and (B) posterior group.** Results are displayed on transversal slices (numbers indicate MNI coordinates) of the ch2better.nii.gz template in MRIcroGL (https://www.nitrc.org). The colour code indicates in how many patients a given voxel was lesioned. The images are displayed in neurological convention (left is left).

Patients’ lesions were classified based on MRI or CT scans obtained as part of their clinical investigation. Source imaging data were available for 187 patients (MRI: *n* = 181, CT: *n* = 6; frontal: *n* = 108, posterior: *n* = 79). MRI scans were acquired on either a 3T or 1.5T Siemen scanner, and CT scans were acquired using spiral CT systems. Lesions were traced and independently classified using MIPAV (https://mipav.cit.nih.gov/) by J.M. and E.C. and checked by P.N., who was blind to the study results. The lesion masks were segmented and non-linearly normalized to Montreal Neurological Institute (MNI) stereotaxic space at 2 × 2 ×2 mm resolution using SPM-12 software (Wellcome Department of Imaging Neuroscience, London, England: http://www.fil.ion.ucl.ac.uk; see^26^ for details). Patients with frontal lesions were identified as those with a lesion in any part of the brain anterior to the central sulcus and superior to the lateral fissure. Patients with posterior lesion were identified as those with a lesion affecting any brain area posterior to the central sulcus and inferior to the lateral fissure (see Robinson *et al*.^6^ for a similar method). Patients were classified using templates based on Brodmann area maps provided with MRIcron (http://www.sph.sc.edu/comd/rorden/mricron). The distribution of patients’ lesions is presented in Fig. 1. For all remaining patients (*N* = 88) whose scans could not be accessed, MRI or CT scans had previously been reviewed by a radiologist as part of routine clinical care. These patients were classified as having frontal or posterior lesions where the radiological report stated that there was evidence of a focal lesion affecting only frontal or posterior areas, respectively, as defined above.

### Behavioural investigations

Premorbid optimal level of functioning was estimated using the NART, and naming was assessed using the GNT. All tests were scored in the published standard manner. Due to the retrospective nature of our study, GNT test data were unavailable for 29% of the sample. Phonemic fluency was assessed by the S fluency test, which required patients to generate as many words as possible starting with the letter S within 60 s. Patients were told not to produce proper nouns, change the ending of words (e.g. ‘eat, eating, eaten’) or repeat words.

#### Errors

Errors were counted when participants broke one of the task rules, ‘rule break errors’, or repeated a word, ‘perseverations’. Specific types of rule break errors were also classified, namely, inappropriate words (e.g. ‘s**t’), proper nouns (e.g. ‘Samantha’) and permutations of previous words (e.g. ‘say, saying’; see Supplementary Material for a more detailed description of how inappropriate words were classified). It should be noted that, although not explicitly stated as a rule, producing inappropriate words in the context of a neuropsychological assessment can be considered a type of error. Hence, following our previous methodology, we classified inappropriate words as rule break errors.^5,9^ Too few words beginning with the wrong letter were produced for meaningful analysis of this type or rule break error. We calculated the percentage of errors produced relative to the total number of words generated for all errors (i.e. number of errors/number of words × 100; see Cipolotti *et al*.^5^ for a similar method).

#### Word frequency

We extracted word frequency values using wordfreq Python library. Wordfreq is based on a large corpus of both spoken and written word from Wikipedia, movie and television subtitles, books, Twitter, and other sources.^27^ To ensure that these ratings were an accurate reflection of word frequency in spoken language, we conducted an additional validation procedure for the purposes of this study (see Supplementary Materials). We classified low-frequency words as those with a word frequency value that fell at least two standard deviations below the mean of the entire sample. We compared groups in terms of the percentage of low-frequency words produced relative to the total number of correct words generated (i.e. number of low-frequency words/number of correct words × 100; see Cipolotti *et al*.^5^ for a similar method). To enable direct comparison of the word frequencies obtained by human rating and those obtained by the wordfreq library, the wordfreq raw values were transformed by multiplying by 10^9^ and then decimal logged; words absent from the dictionary were coded as ‘not a number’.

#### Clustering and switching

Using the method of analysis developed by Troyer and colleagues,^11^ we further characterized responses by classifying sequentially produced words into clusters based on their phonological similarity (e.g. ‘sing, sling’). We calculated mean cluster size^11^ and relative number of switches (i.e. number of switches/(total number of words produced −1) × 100).^28^

### Full sequence analysis

#### Large language model representations and graph modelling

We used the GPT-3 Babbage engine (‘text-similarity-babbage-001’)^29^ to extract 2048-dimensional representations of each participant’s generated word sequence. These representations are determined by the many properties of a word sequence—from the phonological to the semantic—that reflect natural language use, richly captured in a 2048-dimensional space. To enable comparison between the sequences of different patients, we derived the pairwise cosine similarities between the representations as a weighted adjacency matrix, formulating their relation as a graph where each participant sequence is a node and the pairwise similarity is an edge between two nodes.

A Bayesian hierarchical nested stochastic block model (SBM)^30^ was then used to infer characteristic ‘communities’ of participants defined by the differences and similarities in their responses as captured by the graph formulation of their relations. SBMs seek to express a graph succinctly in terms of the similarities and differences between the connectivity patterns within it, enabling formal inference of structured patterns of relations. We included age and NART scores as covariates but not GNT scores, owing to the absence of significant loading on the GNT factor in the preceding analyses. Edges were colour-mapped to the average word frequency of each participant’s set of generated words. Importantly, because the purpose of this analysis was to investigate whether this unsupervised approach could independently identify characteristic patterns in the quality of patients’ responses, categorical descriptions (e.g. manually scored rule break errors) or any other features were not added to the model.

#### Predictive modelling

We quantified the relative contribution of baseline variables (age, NART and fluency score), error variables and GPT-3 representations to predicting frontal involvement, by performing Bayesian logistic regression implemented in BayesReg 1.91^31^ running on MATLAB version (R2021b). We estimated a series of models with the frontal versus healthy control and posterior groups as the binary target. Three models were compared: first, a ‘baseline’ model of age, NART and fluency score; second, an ‘errors’ model of the foregoing features plus rule break error proportions, proportion of low-frequency words, number of switches, proportion of switches and mean cluster size; and third, a ‘full’ model of all features (baseline and errors) plus GPT-3 representations of the patients’ word sequences. Given the relatively small sample size available for prediction, we used the most compact GPT-3 language representation, ‘text-similarity-ada-001’ (1024 dimensions), further reduced to 256 latent variables by random projection. All models were estimated with Markov chain Monte Carlo (MCMC) employing a single chain over 100 000 samples following 100 000 burn-in with thinning of 10. The ‘baseline’ and ‘errors’ models were configured with a ridge prior; the ‘full’ model was configured with a horseshoe prior to promote sparsity. The effective sample size exceeded 99% for all variables across all reported models. Models were compared by their pseudo *R*^2^ value and, to correct for differences in the number of parameters, by the Watanabe–Akaike information criterion (WAIC).^32^

### Statistical analysis

All statistical analyses were conducted using SPSS version 25, except analyses of language representations, which were conducted in Python 3.5. Skewness and kurtosis were assessed by inspecting boxplots, and homogeneity of variances was assessed using Levene’s test.

We compared frontal, posterior and healthy control groups on demographic variables, using ANOVA and Fisher’s exact test, and on neuropsychological test performance, overall S fluency performance and S fluency errors, word frequency and clustering/switching scores using analysis of covariance (ANCOVA), controlling for age and NART scores. Following significant differences, we used *post hoc* tests with Bonferroni correction (alpha 0.05/3 = 0.016) to compare frontal versus posterior, frontal versus healthy control and posterior versus healthy control groups.

We then repeated the analyses described above but this time compared left frontal, right frontal, left posterior, right posterior and healthy control groups. Significant results were followed by Bonferroni-corrected pairwise comparisons using alpha 0.05/4 = 0.0125 to compare each patient group against the healthy control group (i.e. left frontal versus healthy control, right frontal versus healthy control, left posterior versus healthy control, right posterior versus healthy control groups). Pairwise comparisons were then undertaken to compare left versus right frontal, left versus right posterior and left frontal versus left posterior groups.

Statistical comparisons between communities identified on SBM were between the index community and all other communities (one-versus-rest) using Bonferroni-corrected (alpha/40) Mann–Whitney U tests.

## Results

### Demographics and background tests

Frontal, posterior and healthy control groups were well matched for age, ethnicity, gender and years of education (all *P* > 0.05; see Table 1). When the analysis was broken down by laterality, both left frontal and left posterior groups were found to have a significantly higher proportion of males than the healthy control group [*χ*^2^ (1, *n* = 199) = 7.42, *P* < 0.01, *ϕ* = 0.19; *χ*^2^ (1, *n* = 169) = 8.68, *P* < 0.01, *ϕ* = 0.23; respectively]. In terms of aetiology, there was a significantly higher proportion of stroke patients in the posterior than the frontal group [*χ*^2^ (1, *n* = 143) = 3.98, *P* < 0.05, *ϕ* = 0.17] but there was no significant difference between the groups in terms of the proportion of tumour patients. When this analysis was broken down by laterality, the only significant effects were that there was a higher proportion of stroke patients in the left posterior versus left frontal group and a higher proportion of tumour patients in the left frontal versus left posterior group [*χ*^2^ (1, *n* = 96) = 5.56, *P* < 0.05, *ϕ* = 0.24; *χ*^2^ (1, *n* = 96) = 4.66, *P* < 0.05, *ϕ* = 0.22; respectively]. However, there were no significant differences in the proportion of stroke or tumour patients between the left and right frontal or left and right posterior groups. The frontal and posterior groups did not significantly differ in terms of lesion volume. Although the left posterior group had significantly smaller lesion volume than the right posterior group [*t*(73) = −2.51, *P* = 0.02], importantly, there were no other significant differences between groups in terms of lesion volume.

There was no significant difference between tumour and stroke patients for overall S performance or mean time between resection/stroke and neuropsychological assessment [*t*(235) = −0.72, *P* = 0.47; *t*(173) = 1.01, *P* = 0.32, respectively]. The median time between stroke/tumour resection and assessment was 24 days [interquartile range (IQR) = 109]. There were no significant differences between any groups in terms of scores on the NART or GNT (all *P* > 0.05).

### Overall S performance

There was a left frontal effect for the total number of correct words produced on the S fluency test. Hence, the frontal group produced significantly fewer correct words than both posterior and healthy control groups [*F*(2337) = 19.960; *P* < 0.001, ηp^2^ = 0.11; *post hoc* tests both: *P* < 0.001]. Analysis of lateralized effects showed that both the left and right frontal groups produced significantly fewer correct words than the healthy control group (both: *P* < 0.001). Importantly, the left frontal group produced significantly fewer words than the right frontal and left posterior groups (*P* < 0.01; *P* < 0.001; respectively).

#### Errors

The list of generated words on the S fluency test was analysed for rule break errors (specifically inappropriate words, proper nouns and permutations of previous words) and perseverations. We found a significant left frontal effect for rule break errors (Table 2). Thus, the frontal group produced a significantly higher percentage of rule break errors than both posterior and healthy control groups [ANCOVA: *F*(2337) = 11.65; *P* < 0.001, ηp^2^ = 0.07; *post hoc* tests; *P* < 0.01; *P* < 0.001, respectively] and breaking down the analysis by laterality revealed that both left and right frontal groups produced a significantly greater percentage of rule break errors than healthy controls (*P* < 0.001; *P* < 0.01, respectively), with the left frontal group also producing a significantly greater percentage of rule break errors than the right frontal and left posterior groups (*P* < 0.05; *P* < 0.01, respectively).

**Table 2:** Mean percentage of different error types and low-frequency words produced on S fluency

	Frontal			Posterior			HC
	Total frontal	Left frontal	Right frontal	Total posterior	Left posterior	Right posterior	*n* = 136
	*n* = 143	*n* = 63	*n* = 80	*n* = 96	*n* = 33	*n* = 63	
Rule break errors (SD)	**4.37** a** b*** (6.**47)**	**5.40** b*** e* f** (7.**37)**	**3.56** b** (5.**57)**	2.35 (4.14)	1.79 (3.84)	2.64 (4.28)	2.00 (3.53)
Inappropriate words (‘s**t’) (SD)	**1.52** b* (3.**98)**	1.47 (4.04)	1.56 (3.95)	1.00 (2.54)	0.69 (1.92)	1.16 (2.82)	0.46 (1.64)
Proper nouns (e.g. ‘Samantha’) (SD)	**1.88** b* (4.**51)**	**2.66** b*** f* (5.**33)**	1.26 (3.66)	1.21 (3.37)	1.14 (3.62)	1.25 (3.26)	0.80 (2.07)
Permutations (e.g. ‘say, saying’) (SD)	1.20 (3.27)	**1.80** b** e* f* (3.**92)**	0.73 (2.59)	0.65 (2.02)	0.32 (1.28)	0.82 (2.31)	0.81 (2.40)
Perseverations (‘sun, … sun’) (SD)	1.21 (3.09)	1.59 (3.80)	0.90 (2.36)	**2.54** c* (4.**30)**	**3.88** b** d* g* (5.**36)**	1.84 (3.47)	1.73 (2.96)
Percentage of low-frequency words (SD)	**3.98** a* b* (5.**52)**	**4.37** b* (5.**99)**	3.67 (5.12)	2.79 (4.40)	2.30 (4.14)	3.06 (4.54)	2.48 (3.70)

Scores with significant *P* values are in bold.

HC, healthy control; *n*, number; SD, standard deviation.

^a^Significant difference from posterior.

^b^Significant difference from healthy controls.

^c^Significant difference from frontal.

^d^Significant difference from left frontal.

^e^Significant difference from right frontal.

^f^Significant difference from left posterior.

^g^Significant difference from right posterior.

*P < 0.05; **P < 0.01; ***P < 0.001.

Analysis of individual types of rule break error revealed a frontal effect for inappropriate words, with the frontal group producing a significantly greater percentage of inappropriate words than healthy controls [*F*(2337) = 4.41, ηp^2^ = 0.03; *P* < 0.05; *post hoc* test: *P* < 0.05] but with no significant lateralized effects. There was a left frontal effect for proper nouns. Hence, the frontal group produced a significantly greater percentage of proper nouns than healthy controls [*F*(2337) = 4.22, ηp^2^ = 0.02; *P* < 0.05; *post hoc* test: *P* < 0.05] and analysis of lateralized effects showed that the left frontal group produced a significantly greater percentage of proper nouns than the left posterior and healthy control groups (*P* < 0.05; *P* < 0.001, respectively). There was also a left frontal effect for permutations: we found no significant difference between frontal, posterior and healthy control groups; however, when laterality was included in the analysis, we found that the left frontal group generated a significantly greater percentage of permutations than right frontal, left posterior and healthy control groups [*F*(4335) = 2.86, ηp^2^ = 0.03; *P* < 0.05; pairwise comparisons: *P* < 0.05; *P* < 0.05; *P* < 0.01, respectively]. In contrast, there was a left posterior effect for perseverations. Thus, the posterior group produced a significantly higher percentage of perseverations than the frontal group [*F*(2337) = 4.00, *P* < 0.05, ηp^2^ = 0.02; *post hoc* test: *P* < 0.05]. Moreover, the left posterior group produced a significantly greater percentage of perseverations than the left frontal, right posterior, and healthy control groups (*P* < 0.05; *P* < 0.05; *P* < 0.01, respectively).

#### Word frequency

We analysed the frequency of word usage generated on the S fluency test by reference to a large text corpus from Wikipedia, movie and television subtitles, books, Twitter and other sources.^33^ Notably, we found that the frontal group produced a significantly greater percentage of low-frequency words than both posterior and healthy control groups [*F*(2311) = 4.83, *P* < 0.01, ηp^2^ = 0.03; *post hoc* tests: both *P* < 0.05]. Furthermore, the left frontal group produced a significantly greater percentage of low-frequency words than left posterior and healthy control groups [*F*(4309) = 3.30, *P* < 0.05, ηp^2^ = 0.04; pairwise comparisons: *P* < 0.05; *P* < 0.001, respectively].

#### Clustering and switching

We classified sequentially produced words into clusters based on their mean cluster size and relative number of switches (calculated to reduce confounding by total words produced^29^). We found no significant effects for either variable (see Supplementary Material, Supplementary Table 3).

We repeated all the analyses described above (i.e. for demographics, background neuropsychological tests, overall S performance, errors, word frequency and clustering and switching) including only the 187 patients for whom scans could be accessed. Importantly, the pattern of results was overall unchanged (see Supplementary Tables 1 and 2).

### Word sequence analysis

#### Transfer learning and graph modelling

Aiming to capture complex patterns conveyed in the full sequence of correct and incorrect words generated in the phonemic fluency task, we used the GPT-3 Babbage engine (‘text-similarity-babbage-001’) to extract 2048-dimensional representations of each participant’s generated word sequences. We then used a generative model of the modular structure of graphs—a Bayesian hierarchical nested SBM^30^—in an unsupervised manner (blinded to frontal, posterior and healthy control labels) to infer characteristic ‘communities’ of participants defined by the differences and similarities in their responses. Age and NART scores were added as covariates to this model, and the edges were colour-mapped to the average word frequency of each participant’s set of generated words.

**Figure 2:**
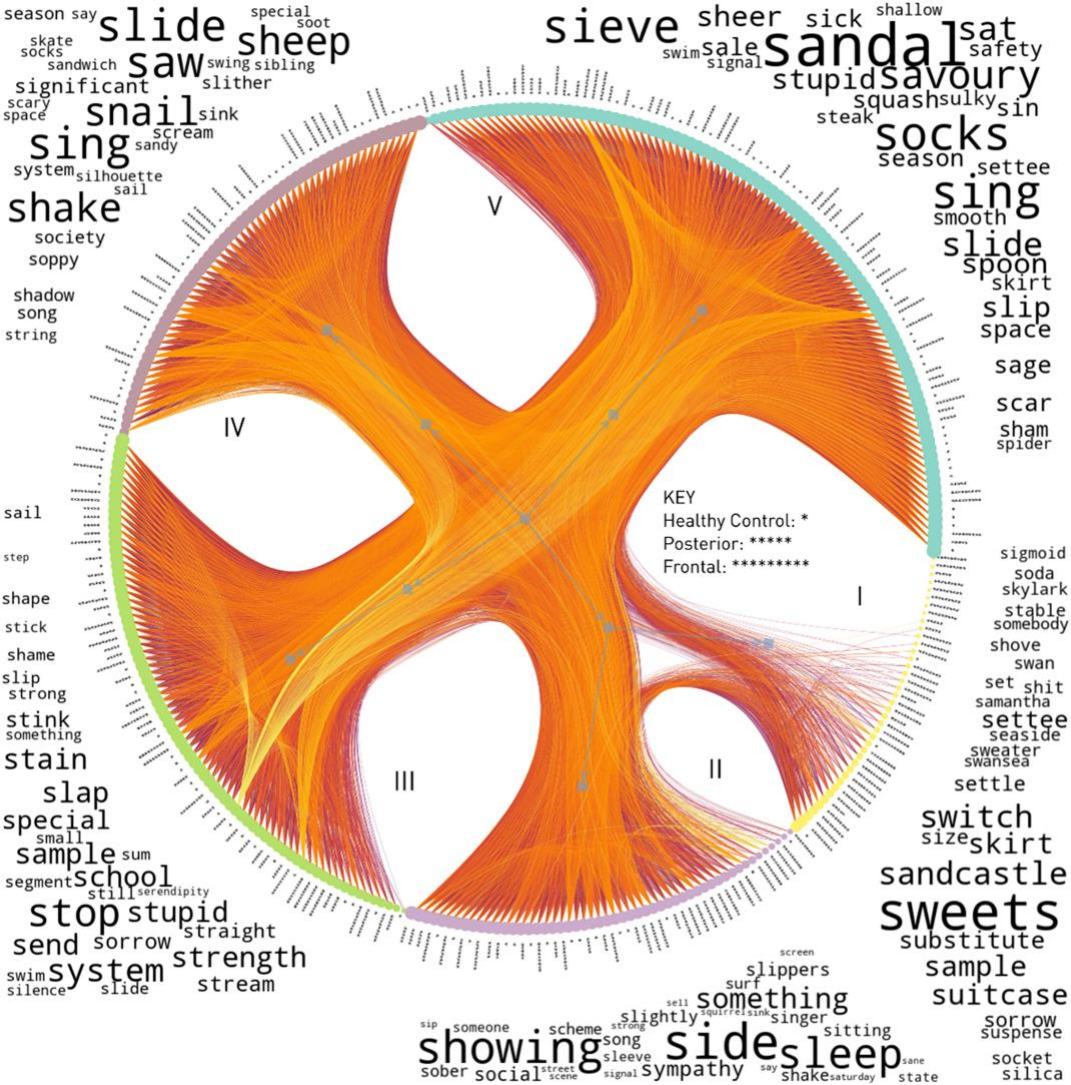
Nested SBM of word sequences produced by healthy controls (*), posterior patients (*****) and frontal patients (*********) on the phonemic fluency task, embedded by the GPT-3 Babbage engine. Graphs encode nodes—here uttered sequences—connected by edges describing the relationship between pairs of nodes; here, the edges are pairwise cosine similarity of representations, with age and NART score as edge covariates. Edge colour is the average rarity of words in the sequence, with increasingly rare scores displayed in darker colour. Word clouds of the most characteristic words generated by each community, quantified by term frequency–inverse document frequency—a measure of the relative prominence of a term in a set of documents—are displayed next to their graph community, with more important words appearing in larger size.

GPT-3 representations of the word sequences generated by frontal, posterior and healthy control groups—analysed by their pairwise cosine similarities, with age and NART as covariates—were clearly separable into five distinct communities, derived from statistical evidence that within-community relationships are more densely connected than those outside-community, by an SBM model blinded to frontal, posterior and healthy control group membership (Fig. 2). Community separation into ‘blocks’ is performed by the nested hierarchical SBM, which employs an agglomerative multilevel MCMC algorithm to infer the best partition. These communities reflect similarities and differences in uttered sequences as captured by the GPT-3 representations derived ‘without’ knowledge of the participant’s clinical state.

Each of the five communities exhibited significant characteristic differences when compared with the other communities (one-versus-rest) by Bonferroni-corrected Mann–Whitney U tests (Table 3). Communities I (75%) and II (43.7%) had the largest percentages of frontal patients. Community I had a significantly larger percentage of left frontal patients than any other community. Both communities exhibited significantly lower overall S performance. Analysis of the most characteristic words produced was obtained by indexing term frequency–inverse document frequency. This is an established measure of the relative prominence of a term in a set of documents. This analysis revealed that the most characteristic words in community I were two different types of rule breaks, inappropriate words and proper nouns, and for the correct words, lower frequency words (Fig. 2); Community II did not show these characteristic words.

**Table 3:** Demographics and overall S fluency performance for each community

Group	*n*	Age (SD)	NART (SD)	Fluency score (SD)	HC (%)	Frontal (%)	Posterior (%)	Left frontal (%)	Right frontal (%)
I	40	51.9 (16.3)	103.0 (12.2)	7.5 (3.9) ***	7.5***	77.5***	15	42.5 ***	35
II	52	50.8 (14.9)	107.0 (11.5)	11.3 (2.2) ***	28.8	48.1	23.1	21.2	26.9
III	77	48.9 (13.0)	112.4 (9.2) **	19.4 (4.3) ***	37.7	27.3	35.1	9.1	18.2
IV	58	29.7 (7.3) ***	105.6 (9.9)	16.8 (3.1)	55.2	32.8	12.1	17.2	15.5
V	99	56.0 (9.3) ***	108.5 (11.2)	16.0 (3.3)	46.5	22.2*	31.3	6.1	16.2

Statistical comparisons are provided by Bonferroni-corrected Mann–Whitney U tests between the index community and all other communities (one-versus-rest). For each community, % reflects the percentage of the community which are in a localization group. Scores with significant *P* values are in bold.

HC, healthy control; *n*, number; NART, National Adult Reading Test; SD, standard deviation.

**P < 0.01; ***P < 0.001.

Community III included a similar percentage of healthy controls and posterior patients and fewer frontal patients. It exhibited significantly higher overall S performance, with the highest scores on fluency (almost three times higher than Community I) and NART. Community IV was dominated by a significantly younger population, with the largest percentage of healthy controls, whose fluency scores were almost two times higher than Community I. Community V contained significantly older, predominantly posterior patients and healthy controls and two times higher fluency scores than Community I. Strikingly, there was no distinct community separation of posterior patients.

#### Predictive modelling

To quantify the relative contribution of baseline variables (age, NART and fluency score), error variables and GPT-3 representations of word sequences to predicting frontal involvement, we performed Bayesian logistic regression. We estimated a series of models with patients with frontal lesions as the binary target versus patients with posterior lesions and healthy controls. Since the models vary in the number of inputs, and therefore parameters, we quantified both the fidelity of the model, by pseudo *R*^2^, and its goodness of fit adjusted for the number of free parameters, by the WAIC (lower values are better).^32^

The ‘baseline’ model, including age, NART and fluency score, achieved a pseudo *R*^2^ of 0.0816 and WAIC of 200.1, indicating a poor fit. The ‘errors’ model, including age, NART, fluency score, rule break errors, low-frequency words, number of switches, proportion of switches and mean cluster size, achieved a pseudo *R*^2^ of 0.1507 and WAIC of 190.33, a substantially better fit, even accounting for the greater number of parameters, as shown by the lower WAIC. The ‘full’ model, which included all predictors, age, NART, fluency score, rule break errors, low-frequency words, number of switches, proportion of switches, mean cluster size and word sequences, achieved a pseudo *R*^2^ of 0.3302 and WAIC of 188.2846, a further substantial increase in the goodness of fit. Rerunning the ‘errors’ model with more strongly sparsity promoting priors (lasso, or horseshoe) did not yield a better WAIC than the ‘full’ model (minimum 189.7). The addition of GPT-3 representations thus produced the best predictive model, taking into account the expansion in the number of model parameters.

## Discussion

To the best of our knowledge, this is the largest and most comprehensive investigation of qualitative aspects of phonemic fluency performance. We conducted a detailed analysis of errors, low-frequency words and clustering/switching in a very large sample of patients with focal, unilateral frontal or posterior lesions and healthy controls. We further analysed patients’ and healthy controls’ responses by adopting a novel approach based on generative modelling of representations derived from ‘transfer learning’ with deep language models trained on large-scale data. In this approach, we used the recently developed GPT-3 Babbage engine to analyse word sequences and fitted unsupervised generative hierarchical graph models to reveal characteristic patterns of performance arising as ‘communities’ within the graph. We then conducted predictive modelling to investigate whether deep language representations of word sequences significantly improved the accuracy of detecting frontal dysfunction using the phonemic fluency test.

Our error analysis revealed, for the first time, significant effects for specific types of rule break error. Thus, there was a significant non-lateralized frontal effect for inappropriate words (e.g. ‘s**t’). We speculate that this may be related to an impairment in self-monitoring processes, necessary to judge the social appropriateness of phonemic output. We found a left frontal effect for proper nouns (e.g. ‘Samantha’). Thus, the frontal group produced a significantly greater percentage of proper nouns than healthy controls and, when the analysis was broken down by laterality, the left frontal group was found to produce a significantly greater percentage of proper nouns than left posterior and healthy control groups. We also found a left frontal effect for permutations (e.g. ‘say, saying’). For these errors, the analysis was only significant when laterality was included. This showed that the left frontal group produced a significantly greater percentage of permutations than not only left posterior and healthy control but also right frontal groups. Notably, permutation errors almost always occurred immediately after the responses from which they were derived (e.g. ‘see, seeing’), suggesting that these errors were somewhat perseverative in nature. Given that the task instructions explicitly state that proper nouns and permutations are forbidden, these errors may reflect a failure to adopt and sustain the task instructions.

Our analysis of correct responses revealed that the frontal group produced a significantly greater percentage of low-frequency words than both posterior and healthy control groups, with the left frontal group producing a significantly greater percentage of low-frequency words than left posterior and healthy control groups. Low-frequency words have fewer associated words than high-frequency words and, hence, elicit less competition with one another. In line with previous suggestions, we hypothesize that the production of low-frequency words may have allowed patients with left frontal lesions to overcome an impairment in the process of selection.^5,33^

In addition to the frontal lobe findings, it is interesting that we also found a left posterior effect for a specific type of error—recurrent perseverations—namely, repetitions following at least one intervening response (e.g. ‘sun, sea, sun’).^34^ Recurrent perseverations in naming tasks have been hypothesized to be associated with decreased acetylcholine due to left posterior lesions. Our study provides further evidence of an association between left posterior lesions and recurrent perseverations, on a test of phonemic fluency.^35,36^

Our novel transfer learning approach uses LLM-derived embeddings to enable the analysis of a richer set of characteristics of phonemic fluency responses, incorporating not merely individual words but entire word sequences. Bayesian stochastic block modelling of the relations between embeddings reveals distinct communities of patients with similar word sequences. This unsupervised approach formalizes the task of clustering embedded responses based on their similarities and differences within a principled graph framework of their relations, employing Bayesian inference to maximize robustness and statistical efficiency. It permits formal inference to the presence or absence of community structure by model comparison founded on the models’ minimum description length. Two distinct communities (I and II) enriched in patients with frontal lesions emerged, both exhibiting significantly lower overall S performance. Crucially, the most characteristic words in Community I tended to be inappropriate words, proper nouns and low-frequency words. This is notable, given that this community was dominated by patients with left frontal lesions. Note that the comparatively modest number of patients in each group precludes high-resolution anatomical analysis or finer behavioural subdivision. Overall, these results strongly converged with those obtained by our single word analysis of errors and low-frequency words.

In our predictive modelling analysis, we investigated whether analysis of word sequences significantly improved the accuracy of detecting frontal lobe dysfunction. To this end, we constructed three models and compared their ability to detect the presence or absence of frontal lesions. The first of these, a ‘baseline’ model (including age, NART and overall S performance), was found to significantly predict frontal damage. The second, an ‘errors’ model, included the variables in the baseline model but also included rule break errors, low-frequency words and clustering/switching. This model predicted frontal lobe damage more accurately than the baseline model. The third ‘full’ model included all preceding variables but also included GPT-3 representations of the word sequences. Strikingly, this model was a far more accurate predictor of frontal damage than either of the first two models. Of note, the full model explained approximately four times the variance accounted for by the baseline model and over twice the variance accounted for by the errors model. These findings offer a compelling demonstration that our transfer learning approach can be used to leverage latent, yet diagnostically valuable, characteristics of phonemic fluency responses not exploited by traditional methods of analysis. Combined with a fluency test administered in digital form—for example, with automated transcription of the patient’s responses—our model could enable immediate behavioural phenotyping, providing priors for subsequent anatomical localization. It could also enable finer phenotypic stratification of patients, facilitating more accurate monitoring of disease evolution at the individual level and more robust inference of prognosis and interventional effects at the group level.

The implications of our findings extend beyond the analysis of performance on the phonemic fluency test and are of relevance to focal lesion studies more generally. The quality of responses produced by patients with focal brain lesions is held to be unique as a source of evidence.^1,2^ Our findings demonstrate, however, that the wealth of potentially valuable information that focal lesion studies provide is not adequately captured using traditional methods of analysis. The current investigation was necessarily limited in scope to analysis of performance on one task, and in the interest of producing a cohesive and focused study, we opted to analyse only the impact of word frequency on language production. Future studies including concreteness of words may provide interesting insights into the lateralization of the mechanisms involved in language production and could enrich diagnosis via the machine learning model.

Nonetheless, our novel methodological approach offers a promising proof of concept and that may be applied to analysis of performance on tests used to investigate other cognitive domains. One notable example is human intelligence, where current understanding is limited by the necessity to reduce complex behaviour to that which is measurable using simplistic measures of performance.

In conclusion, our findings demonstrate the significant inferential and diagnostic value of characterizing qualitative features of phonemic fluency performance using deep language modelling. We have introduced a novel transfer learning and graph modelling methodology that offers a promising approach to the analysis of qualitative aspects of performance on cognitive tests.

## Supplementary material

Supplementary material is available at *Brain Communications* online.

## Supplementary Material

fcad318_Supplementary_Data

## Data Availability

The behavioural data and code that support the findings of this study are available from L.C., upon reasonable request, or are freely available here: https://github.com/high-dimensional.
