# Supplementary material for: Characterizing phonemic fluency by transfer learning with deep language models
Source: Brain Commun. 2023 Nov 28;5(6):fcad318. doi: 10.1093/braincomms/fcad318 (PMC10691875; doi:10.1093/braincomms/fcad318)
Supplement: fcad318_Supplementary_Data [file fcad318_supplementary_data.docx]

**Supplementary Table 1 Demographics and overall S fluency performance in 187 patients for whom scans were available.**

|  | **Frontal** | | |  | **Posterior** | | | **HC** |
| --- | --- | --- | --- | --- | --- | --- | --- | --- |
|  | **Total Frontal**  ***n* = 108** | **Left Frontal**  ***n* = 45** | **Right Frontal**  ***n* = 63** |  | **Total Posterior**  ***n* = 79** | **Left Posterior**  ***n* = 25** | **Right Posterior**  ***n* = 54** | ***n* = 136** |
| Age (years)  (SD) | 47.45  (16.26) | 45.18  (15.38) | 49.08  (14.64) |  | 50.85  (14.17) | 50.48  (16.63) | 51.02  (13.05) | 47.93  (15.38) |
| Gender (Male/Female) | **61/47 b*** | **28/17 e**** | 33/30 |  | **45/34 b**** | **16/9 g**** | 29/25 | 60/76 |
| Ethnicity (White British/Black African or Caribbean/Asian Indian/Other) | 86/2/3/11 | 34/1/1/4 | 52/1/2/7 |  | 66/1/4/3 | 24/0/0/1 | 42/1/4/2 | - |
| Aetiology (stroke/tumour/abscess) | **21/86/1 a*** | 4/40/1 | **17/46/0 d*** |  | 26/53/0 | 9/16/0 | 17/37/0 |  |
| Education (years)  (SD) | 14.07  (3.51) | 13.92  (3.43) | 14.17  (3.41) |  | 13.60  (3.05) | 13.87  (3.06) | 13.45  (3.06) | 13.77  (2.60) |
| Lesion volume (mm^3^)  (SD) | 44.17  (48.66) | 48.19  (46.85) | 41.15  (45.63) |  | 49.69  (69.00) | **28.60 g***  **(27.29)** | 60.23  (80.52) |  |
| Premorbid NART IQ  (SD) | 107.79  (12.55) | 105.48  (11.50) | 109.44  (10.49) |  | 108.55  (11.77) | 108.30  (14.00) | 108.67  (10.66) | 108.09  (9.80) |
| GNT (Correct /30)  (SD) | 21.15  (3.47) | 20.28  (3.50) | 21.80  (3.42) |  | 21.18  (4.25) | 20.50  (4.75) | 21.51  (4.00) | 22.29  (3.57) |
| Overall S performance  (SD) | **13.12 a** b*****  **(5.69)** | **11.73 b*** e* f****  **(5.84)** | **14.11 b*****  **(5.79)** |  | 15.23  (4.44) | 14.92  (4.07) | 15.37  (4.63) | 16.67  (4.05) |

Legend. HC = Healthy Control; *n* = Number; SD = Standard Deviation; NART= National Adult Reading Test; GNT = Graded Difficulty Naming Test. Scores with significant p values are in bold. *= p <0.05; **= p <0.01; ***= p <0.001. Ethnicity was unavailable for 7 patients and all Healthy Controls.

a indicates significant difference from Posterior.

b indicates significant difference from Healthy Controls

d indicates significant difference from Left Frontal.

e indicates significant difference from Right Frontal.

g indicates significant difference from Right Posterior

**Supplementary Table 2 Mean percentage of different error types and low frequency words produced on S fluency in 187 patients for whom scans were available.**

|  | **Frontal** | | |  | **Posterior** | | | **HC** |
| --- | --- | --- | --- | --- | --- | --- | --- | --- |
|  | **Total Frontal**  ***n* = 108** | **Left Frontal**  ***n* = 45** | **Right Frontal**  ***n* = 63** |  | **Total Posterior**  ***n* = 79** | **Left Posterior**  ***n* = 25** | **Right Posterior**  ***n* = 54** | ***n* = 136** |
| Rule break errors  (SD) | **5.18 a*** b*****  **(6.80)** | **6.43 b*** f****  **(7.69)** | **4.29 b*****  **(6.00)** |  | 2.62  (4.39) | 2.14  (4.24) | 2.85  (4.47) | 2.00  (3.53) |
| -Inappropriate words (“*s**t*”)  (SD) | **1.84 b****  **(4.45)** | **1.83 b***  **(4.63)** | **1.84 b***  **(4.35)** |  | 1.09  (2.70) | 0.91  (2.17) | 1.17  (2.92) | 0.46  (1.64) |
| -Proper nouns (e.g. “*Samantha*”)  (SD) | **2.19 b****  **(4.74)** | **3.14 b*** f***  **(5.48)** | 1.51  (4.04) |  | 1.40  (3.64) | 1.28  (4.03) | 1.46  (3.48) | 0.80  (2.07) |
| -Permutations (e.g. “*say, saying*”)  (SD) | 1.46  (3.61) | **2.20 b** e* f***  **(4.36)** | 0.93  (2.89) |  | 0.70  (2.10) | 0.42  (1.46) | 0.83  (2.34) | 0.81  (2.40) |
| Perseverations (“*sun, ….. sun*”)  (SD) | 1.16  (3.09) | 1.65  (3.95) | 0.81  (2.26) |  | 2.10  (3.98) | 3.11  (5.35) | 1.63  (3.12) | 1.73  (2.96) |
| Percentage of low frequency words  (SD) | **4.51 a* b****  **(5.56)** | **4.84 b*** f****  **(5.72)** | 4.27  (5.48) |  | 2.91  (4.28) | 1.90  (3.10) | 3.37  (4.68) | 2.48  (3.70) |

Legend. HC = Healthy Control; *n* = Number; SD = Standard Deviation; Scores with significant p values are in bold. *= p <0.05; **= p <0.01; ***= p <0.001

a indicates significant difference from Posterior.

b indicates significant difference from Healthy Controls

e indicates significant difference from Right Frontal.

f indicates significant difference from Left Posterior.

**Supplementary Table 3 Mean cluster size and relative number of switches on S fluency.**

|  | **Frontal** | | |  | **Posterior** | | | **HC** |
| --- | --- | --- | --- | --- | --- | --- | --- | --- |
|  | **Total Frontal**  ***n* = 143** | **Left Frontal**  ***n* = 63** | **Right Frontal**  ***n* = 80** |  | **Total Posterior**  ***n* = 96** | **Left Posterior**  ***n* = 33** | **Right Posterior**  ***n* = 63** | ***n* = 136** |
| Mean cluster size (e.g. “*snore, snail, show*” cluster size =1)  (SD) | 0.35  (0.45) | 0.36  (0.55) | 0.34  (0.37) |  | 0.31  (0.33) | 0.28  (0.29) | 0.32  (0.35) | 0.38  (0.48) |
| Relative number of switches ((number of switches/(total words produced-1)x100)  (SD) | 72.92  (22.02) | 72.71  (26.26) | 73.46  (18.27) |  | 77.20  (14.62) | 78.17  (15.05) | 76.68  (14.47) | 74.32  (17.35) |

Legend. HC = Healthy Control; *n* = Number; SD = Standard Deviation.

**Social appropriateness and spoken word frequency ratings**

For every word generated by Frontal, Posterior and Healthy Control groups during the S fluency task, we obtained subjective ratings of social appropriateness and spoken word frequency. The former ratings were used to classify inappropriate words. The latter ratings were used to ensure that word frequency values extracted from the wordfreq Python library accurately reflected word frequency in spoken language.

We recruited an additional group of 16 healthy volunteers to act as independent raters, matched as closely as possible to the Frontal, Posterior and Healthy Control groups for age (Mean=47.33; SD=18.99), gender (Males 7, Females 8) and years of education (Mean=15.62; SD=2.26). Data from one participant was lost due to a technical error.

Each independent rater was provided with an audio recording of a native English speaker reading the entire corpus of words generated by the Frontal, Posterior and Healthy Controls during the S fluency task (*n*=1354). The same audio recordings were used to obtain social appropriateness and spoken word frequency ratings. Seven of the independent raters completed social appropriateness ratings first while eight completed subjective word frequency ratings first. Importantly, for each rater words were presented in a different fixed semi-randomised for social appropriateness and word frequency ratings and the order of the words was semi-randomised between independent raters. Independent raters were blind to whether each word was produced by Frontal, Posterior or Healthy Control group members.

To obtain social appropriateness ratings, independent raters were asked to rate each word on a scale from 0 to 3 in terms of how socially appropriate it would be to say that word to a healthcare professional during a cognitive assessment (0=‘socially appropriate’, 1=‘somewhat socially inappropriate’, 2=‘very socially inappropriate’, 3=‘extremely socially inappropriate’). We focused our analysis on words rated by the majority of independent raters as socially inappropriate (rating >0). To obtain spoken word frequency ratings, independent raters were asked to rate each word on a scale from 0 to 6 in terms of its subjective word frequency in spoken language (0=‘unknown’, 1=‘extremely infrequent’, 2=‘very infrequent, 3=‘somewhat infrequent’, 4=‘somewhat frequent’, 5=‘very frequent’, 6=‘extremely frequent’). For each word, we averaged the ratings to calculate a measure of subjective spoken word frequency. For both social appropriateness and subjective spoken word frequency ratings, intraclass correlations were significant (both *p*<0.001) and indicated excellent inter-rater reliability (0.914, 0.948, respectively).

Spoken word frequency ratings were used for the purposes of validating the word frequency values extracted from the wordfreq Python library. We considered the latter values preferable for use in the main analyses, given that they had a wider distribution, making them potentially more sensitive to potential word frequency effects. We found a strong, significant positive correlation between values extracted from the wordfreq Python library and those obtained from the group of independent raters, suggesting that the word frequency values used for the main analysis were an accurate reflection of word frequency in spoken language (r = 0.75; p <0.001; see Supplementary Figure 1).


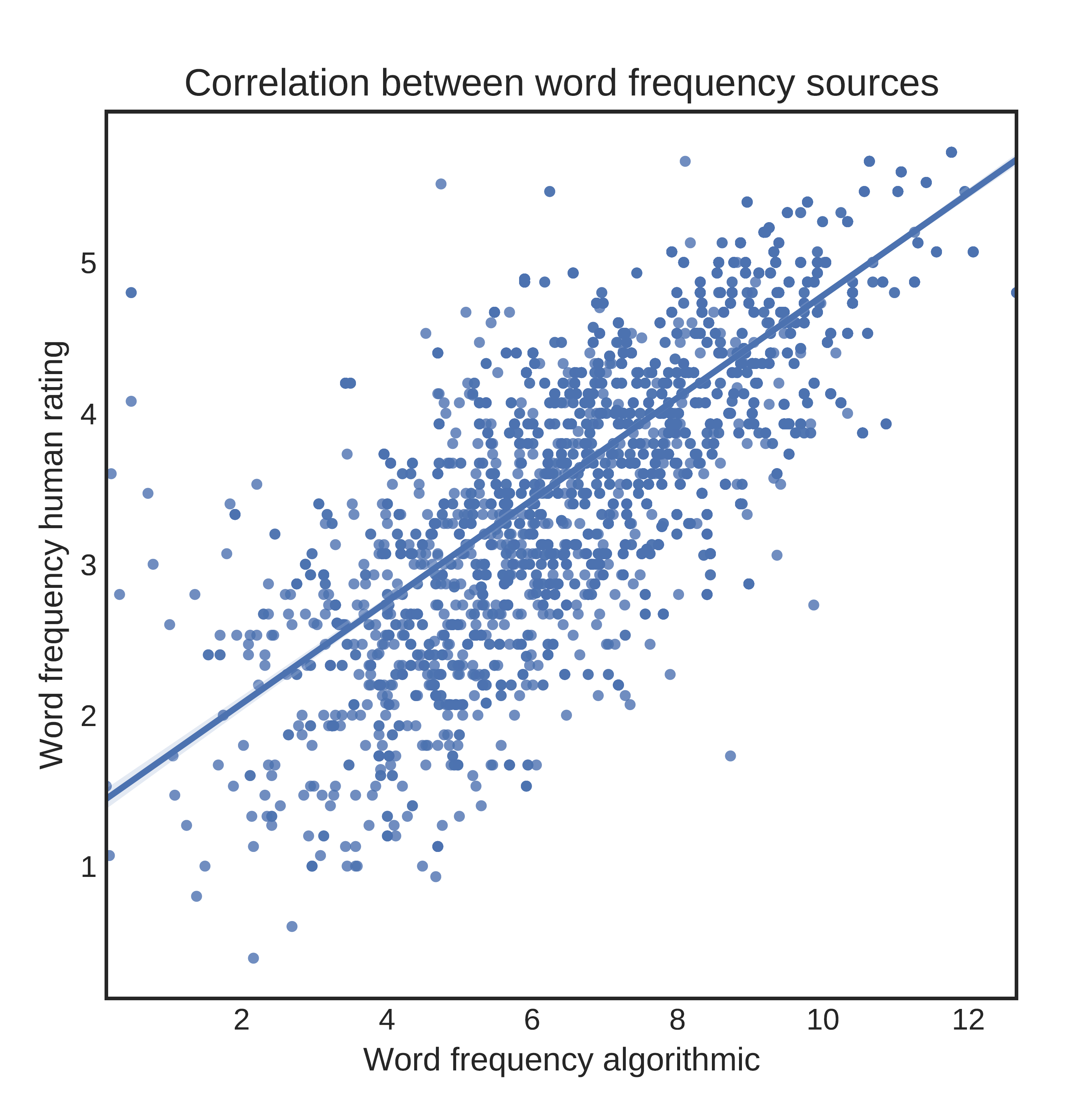


**Supplementary Figure 1.** Scatterplot of spoken word frequency ratings for the entire corpus of words generated by the Frontal, Posteriors and Healthy Control groups during the S fluency task (*n*=1354). Spoken word frequency ratings extracted from the wordfreq Python library are shown on the X axis and Spoken word frequency ratings obtained from the group of independent raters are shown on the Y axis.
